# Supplementary material for: From Peak to Plunge: A Multi-Database Analysis of State-Level Disparities in Hydromorphone Use in the US
Source: Pharmacy (Basel). 2025 Oct 13;13(5):147. doi: 10.3390/pharmacy13050147 (PMC12567197; doi:10.3390/pharmacy13050147)
Supplement: Supplementary file 1 [file pharmacy-13-00147-s001.zip › Supplemental Table 1 Highest and lowest .pdf]

Supplemental Table S1: States with the highest and lowest Medicaid prescriptions/thousand adult population in 2023.

Highest States	Medicaid hydromorphone prescriptions/thousand adult population
1.Delaware	14.88
2. Virginia	7.10
3. Idaho	6.61
4. North Dakota	6.43
5. Oregon	5.31
6. Vermont	5.11
7. Montana	5.02
8. Iowa	4.78
9. Kentucky	4.69
10. New Hampshire	4.57
**Lowest States**	**Medicaid hydromorphone prescriptions/thousand adult population**
42. Texas	0.79
43. Wisconsin	0.70
44. Utah	0.70
45. Hawaii	0.70
46. Illinois	0.62
47. Michigan	0.56
48. Arkansas	0.48
49. Oklahoma	0.48
50. Wyoming	0.43
51. South Carolina	0.42
