# Supplementary figures and images for: From Peak to Plunge: A Multi-Database Analysis of State-Level Disparities in Hydromorphone Use in the US
Source: Pharmacy (Basel). 2025 Oct 13;13(5):147. doi: 10.3390/pharmacy13050147 (PMC12567197; doi:10.3390/pharmacy13050147)

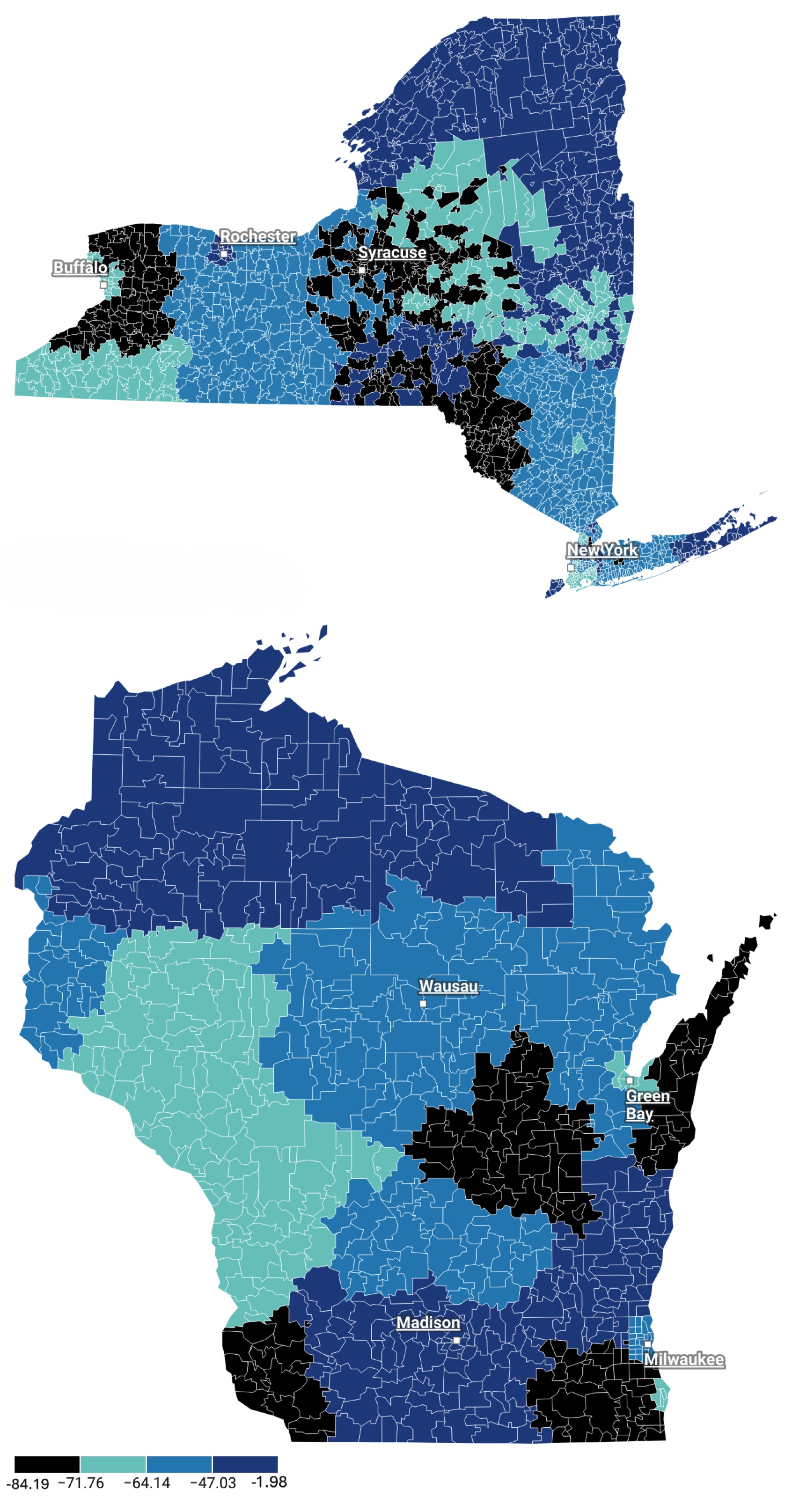

Supplement: Supplementary file 1 [file pharmacy-13-00147-s001.zip › hopeful final ny and wis.jpg]
